# CircSPI1 acts as an oncogene in acute myeloid leukemia through antagonizing SPI1 and interacting with microRNAs

**DOI:** 10.1038/s41419-021-03566-2

**Published:** 2021-03-19

**Authors:** Xiaoling Wang, Peng Jin, Yi Zhang, Kankan Wang

**Affiliations:** 1 Shanghai Institute of Hematology, State Key Laboratory of Medical Genomics, National Research Center for Translational Medicine at Shanghai, Ruijin Hospital Affiliated to Shanghai Jiao Tong University School of Medicine and School of Life Sciences and Biotechnology, Shanghai Jiao Tong University, Shanghai, China; 2 Sino-French Research Center for Life Sciences and Genomics, Ruijin Hospital, Shanghai Jiao Tong University School of Medicine, Shanghai, China

**Keywords:** Mechanisms of disease, Transcriptional regulatory elements

## Abstract

PU.1 (encoded by *SPI1*) is essential for myeloid development, and inhibition of its expression and activity can lead to acute myeloid leukemia (AML). The precise regulation of PU.1 expression is crucial for the development of AML, and the discovery of circular RNAs (circRNAs) can add a new layer of information on regulation. Here, we found that circSPI1, the circular RNA derived from the *SPI1* gene, is highly expressed in AML but not in normal counterparts. Unlike SPI1, a tumor suppressor and being lowly expressed in AML, we demonstrate that circSPI1 acts as an oncogene, evidenced by the observation that circSPI1 knockdown induces myeloid differentiation and apoptosis of AML cells. We provide mechanistic evidence for multiple regulatory roles of circSPI1 in AML progression. On one hand, circSPI1 contributes to myeloid differentiation of AML cells by interacting with the translation initiation factor eIF4AIII to antagonize PU.1 expression at the translation level. On the other hand, circSPI1 contributes to proliferation and apoptosis by interacting with miR-1307-3p, miR-382-5p, and miR-767-5p; this role is uncoupled with SPI1. Finally, we illustrate the clinical significance of circSPI1 by showing that circSPI1-regulated genes are associated with the clinical outcome of AML patients. Our data provide new insight into the complex *SPI1* gene regulation now involving circSPI1.

## Introduction

Acute myeloid leukemia (AML) is characterized by the rapid proliferation of immature myeloid progenitors blocked at the various stages of myeloid differentiation^1^. At the molecular level, AML is a heterogeneous genetic disease caused by distinct mutations and translocations, and these genetic alterations commonly cause dysregulation of transcription factors that are critical for the control of myeloid progenitor cell expansion and lineage determination^2^. Mounting evidence has demonstrated the critical importance of PU.1, encoded by the *SPI1* gene, in the pathogenesis of AML^3,4^. The impairment of PU.1 expression or activity has been described in AML with PML/RARα^5^, RUNX1/ETO^6^, FLT3-ITD^7^, and complex karyotype^8^. More interestingly, moderate PU.1 inhibition is common in AML patients^9^ and further studies reveal that the tight control of PU.1 expression or activity is essential for myeloid differentiation, and even graded reduction of PU.1 is critical for AML development^10^. Indeed, mechanistic investigations have demonstrated that PU.1 expression is precisely controlled by multiple mechanisms at the *SPI1* locus, including the promoter region, upstream regulatory elements^11,12^, and noncoding antisense RNAs originating from an intronic promoter^13^. However, much remains to be learned about the regulatory elements at the *SPI1* locus and their contribution to the pathogenesis of AML.

More recently, circular RNAs (circRNAs), a group of functional molecules formed by a covalently closed circular continuous loop, have emerged as important regulators in gene regulation. CircRNAs are highly conserved, stable, and often exhibit tissue- or developmental-stage-specific expression^14^. Recent research has revealed that circRNAs are involved in the carcinogenesis and development of various cancers by sponging microRNA^15^, binding to RNA-binding protein^16^, or translating into peptides^17^. Benefiting from high-throughput RNA-sequencing and bioinformatics technology, numerous circRNAs have been identified to be dysregulated in leukemic cells, but our understanding of circRNA roles in leukemia is limited. In AML, circRNAs, derived from the pathogenesis-related genes, e.g., fusion drivers (PML/RARα and MLL/AF9)^18^, cell cycle genes (MYBL2)^16^, and epigenetics remodelers (ASXL1)^19^, have shown oncogenic effects in accelerating the development of leukemia. However, the function of circRNAs derived from lineage-determining genes commonly dysregulated in AML, such as the *SPI1* gene, remains largely unknown.

In this study, we demonstrated that circular molecules derived from the *SPI1* gene were specifically upregulated and contributed to the leukemogenesis of AML. Unexpectedly, we found that circSPI1 expression and its contributions to myeloid differentiation and apoptosis were antithetical with its linear SPI1. Mechanistically, circSPI1 blocked differentiation through antagonizing PU.1-regulated genes and controlled proliferation and apoptosis by sponging miR-1307-3p, miR-382-5p, and miR-767-5p. We also defined a network of circSPI1-regulated genes that can be collectively used as a prognostic biomarker for AML. Our study provided new insight into the oncogenic activity of circSPI1 in AML and the associated mechanisms in leukemogenesis.

## Materials and methods

### Cell culture

Human AML cell lines THP-1, NB4, and HL60 were maintained in RPMI 1640 medium (HyClone, South Logan, UT) containing 10% fetal bovine serum (Moregate BioTech, Brisbane, QLD, Australia). HEK-293T cells were maintained in RPMI DMEM medium (HyClone) containing 10% fetal bovine serum (Moregate BioTech). All cell lines were cultured at 37 °C in a humidified atmosphere with 5% CO_2_. NB4, THP-1, and HL60 cells were authenticated by karyotype, morphology, and RNA-seq analysis. HEK-293T cells were identified by morphology and capability of virus production. All cell lines were detected to be mycoplasma-free using the One-step Quickcolor mycoplasma detection Kit (Shanghai Yise Medical Technology, Shanghai, China).

### RNA extraction, RNase R treatment, and cytoplasm/nucleus fractionation

Total RNA from THP-1, NB4, and HL60 cells was extracted using the RNeasy Protect Mini Kit (Qiagen, Valencia, CA, USA) according to the manufacturer’s instruction. One μg of total RNA was treated with 2 U of RNase R (Epicentre, Madison, WI, USA) for 30 min at 37 °C, then directly reversed into cDNA using the PrimeScript™ RT reagent Kit with gDNA Eraser (Perfect Real Time) (Takara, Osaka, Japan). Cytoplasm and nucleus RNA were fractionated using the PARIS kit (Thermo Fisher Scientific, Waltham, MA) according to the manufacturer’s instruction.

### RT-PCR, RT-qPCR, and TaqMan probe-based RT-qPCRs

For RT-PCR assays, genes were amplified using the Phanta Max Super-Fidelity DNA polymerase (Vazyme, Nanjing, China). RT-qPCR assays were performed using TB Green^®^ Premix Ex Taq™ (Tli RNaseH Plus) (Takara) on ABI ViiA 7 Real-Time PCR System (Applied Biosystems, Foster City, CA, USA). The relative expression level of a gene was calculated as △△Ct. GAPDH was used as an internal control. TaqMan probe-based RT-qPCR assays were conducted using Premix Ex Taq^TM^ (Probe qPCR) Bulk (Takara). The probes were synthesized from Generay Biotech (Shanghai, China). Sequences of probes and primers are listed in Supplementary Table 1.

### Knockdown experiments

For the production of lentiviruses, 15 μg of the shcircSPI1 or scramble plasmid, 9 μg of the psPAX2, and 6 μg of the pMD2G packaging plasmids were mixed in 45 μL Lipofectamine 2000 (Invitrogen, Carlsbad, CA, USA) and then transfected into HEK-293T cells. The lentiviruses were harvested 48 h after transfection. THP-1 and NB4 cells were infected using lentiviruses with 8 μg/mL polybrene (Sigma, St. Louis, MO, USA) by centrifuging for 90 min at 32 °C at 1200 × *g*. Cells infected with lentiviruses for the indicated days were harvested and used for functional analysis.

### CCK8 proliferation assay

Cells infected with shcircSPI1 or scramble lentiviruses for two days were seeded into a 96-well plate at the concentration of 2000/well. Cell proliferation was measured every day using the CCK8 reagent (Dojindo, Tokyo, Japan) according to the manufacturer’s instruction. Every group of the experiment was conducted in triplicate.

### Flow cytometry analysis

Annexin-APC and PI (Thermo Fisher Scientific) were used for apoptosis analysis. CD11b-PE (Beckman Coulter, Tokyo, Japan) was used for granulocytic differentiation analysis. CD14-Alexa Fluor (Thermo Fisher Scientific) was used for monocytic cell differentiation. Cells were incubated for 15 min with indicated antibodies and then detected using LRSII (BD Biosciences, San Jose, CA). Results were analyzed using the FlowJo software (version 9.3.2).

### Wright–Giemsa staining

Cells infected with shcircSPI1 or scramble lentiviruses for four days were harvested and washed with ice-cold PBS. About 10,000 cells were centrifuged onto a glass slide by cytospin for 5 min at 600 rpm. The glass slides were dried and stained using the Wright–Giemsa staining solution (Sangon Biotech, Shanghai, China) according to the manufacturer’s instruction. Images were obtained from the Leica DM6000B microscope (Leica Microsystems, Wetzlar, Germany) with the objective at ×40 magnification.

### RNA-sequencing (RNA-seq) and analysis

Library preparation was performed with the TruSeq Stranded mRNA kit (Illumina), and libraries were sequenced on a NextSeq 500 in paired-end mode. The adapter sequences were removed using Trim-Galore, and the clean reads were aligned with STAR^20^ and quantified with HT-Seq^21^ using the GRCh38 human assembly. The differential expression analysis was performed using the edgeR package^22^. To identify the differentially expressed genes (DEGs), we used a *P* value of 0.05 and a log_2_ | fold change | > 1. Gene set enrichment analysis (GSEA) was performed utilizing the GSEA method^23^. For the identification of circRNAs, the output of STAR was analyzed with DCC^24^ to detect, filter, and annotate circRNA. Junction read counts were normalized to reads per million mapped reads (RPM). The RNA-seq data were available at NCBI with the GEO accession number GSE159931.

### Western blotting

Cells infected with shcircSPI1 or scramble lentiviruses for 4 days were harvested and lysed in the RIPA high lysis buffer (Beyotime Biotechnology, Shanghai, China). Total protein was loaded and separated on 10% acrylamide tris-HCL-buffered SDS-PAGE gels (EpiZyme, Shanghai, China), transferred to polyvinylidene difluoride membranes (GE Healthcare, Piscataway, NJ, USA), immunoblotted with PU.1 antibody (ab76543, Abcam, Cambridge, MA), eIF4AIII antibody (17504-AP, Proteintech, Wuhan, China), p-ERK1/2 antibody (4370S, Cell Signaling Technology, Beverly, MA), ERK1/2 antibody (4695S, Cell Signaling Technology), CDK6 antibody (3136P, Cell Signaling Technology), BCL2 antibody (3498S, Cell Signaling Technology), and GAPDH antibody (60004-1-Ig, Proteintech). Images were obtained using Immobilon Western Chemiluminescent HRP Substrate (Millipore, Billerica, MA, USA) on the Amersham Imager 600 Luminescent Image analyzer (GE Healthcare Bio-Sciences).

### RNA pulldown assay

The RNA pulldown assay was performed in NB4 cells using the RNA pulldown Kit (BersinBio, Guangzhou, China) according to the manufacturer’s instruction. Briefly, the biotin-labeled circSPI1 probe or negative control probe was incubated with streptavidin magnetic beads. A total of 2 × 10^7^ NB4 cells were collected and lysed in RIP buffer to obtain whole protein extract. The probe magnetic beads and cell extract were mixed and washed with NT2 buffer. Protein bound with the probe was eluted with the protein elution buffer and subjected to western blotting.

### Luciferase assay

HEK-293T cells were plated into 96-well plates at a density of 2 × 10^4^ cells per well for 24 h before transfection. The mixture of the circSPI1 luciferase reporter plasmid, miRNA mimics, and the pRL-SV40 plasmid was co-transfected into HEK-293T cells using Lipofectamine 2000 (Invitrogen). Cells were harvested after transfection for 24 h, and then the luciferase activity assay was performed by the dual-luciferase reporter assay system (Promega, Madison, WI).

### Functional enrichment analysis

Functional enrichment analysis of SPI1/circSPI1-regulated genes was performed using Metascape to group genes into functional pathway terms, including KEGG pathways, GO biological processes, and Hallmark gene sets.

### Prognostic signature generation

The circSPI1-mediated prognostic signature was generated using the penalized LASSO Cox regression with tenfold cross-validation implemented in the glmnet R package. The gene risk score for each patient was calculated using the regression coefficients and gene expression value in the training cohort (TCGA-AML). The same coefficients of each gene were used to calculate the risk score in the validation set (BeatAML). Performances of prognostic signature were assessed by the AUC value under the time-dependent receiver-operating characteristic curve using the survivalROC package. Patients were divided into two groups using the median risk score, and the differences between the two groups were assessed by the log-rank test implemented in the Survival package.

### Statistical analysis

For comparisons of two groups, the statistical significance was estimated using Wilcoxon rank-sum test. For comparisons of more than two groups, the Kruskal–Wallis test was used. For RT-qPCR, the difference for the two groups was calculated using the paired-samples two-tailed Student’s *t* test. Data were reported as mean ± SD. The correlation coefficient was computed using the Pearson method. All statistical analyses were performed using the R/Bioconductor statistical environment (https://www.r-project.org/) and significance was defined as *P* < 0.05.

## Results

### Identification and characterization of circSPI1 in AML

**Fig. 1:**
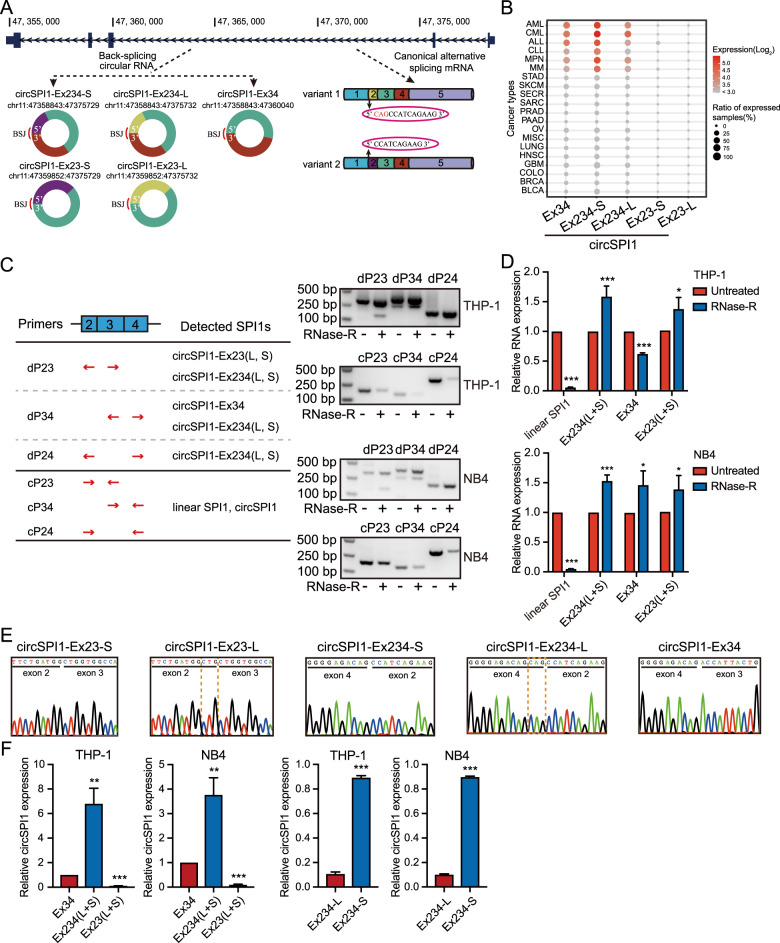
Identification and characterization of circSPI1 in AML. **A**. Schematic overview showed circSPI1 isoforms in *SPI1* locus. Five circSPI1s were retrieved from the circRNA compendium MiOncoCirc database. BSJ back-splicing junction. **B** CircSPI1s were hematopoietic lineage-specific circRNAs. The expression of five circSPI1 isoforms was analyzed in diverse cancer types from MiOncoCirc database. AML acute myeloid leukemia, CML chronic myeloid leukemia, ALL acute lymphocytic leukemia, CLL chronic lymphocytic leukemia, MPN myeloproliferative neoplasm, MM multiple myeloma, STAD stomach adenocarcinoma, SKCM skin cutaneous melanoma, SECR secretory cancer, SARC sarcoma, PRAD prostate adenocarcinoma, PAAD pancreatic adenocarcinoma, OV ovarian serous cystadenocarcinoma, MISC miscellaneous cancer, LUNG lung adenocarcinoma, HNSC head and neck squamous cell carcinoma, GBM glioblastoma multiforme, COLO colon adenocarcinoma, BRCA breast invasive carcinoma, BLCA bladder urothelial carcinoma. **C** The existence of circSPI1s was validated with divergent and convergent primers in THP-1 and NB4 cells. The schematic diagram illustrated the location of divergent and convergent primers and the recognized circSPI1s of divergent primers (left panel), then circSPI1s were detected by RT-PCR (right panels) with divergent and convergent primers before and after treatment with RNase R. Two major bands produced by the divergent primer pair dP23 corresponded to circSPI1-Ex234 (L and S) and circSPI1-Ex23 (L and S), respectively. Two major bands produced by the divergent primer pair dP34 corresponded to circSPI1-Ex234 (L and S) and circSPI1-Ex34. One band produced by dP24 corresponded to circSPI1-Ex234 (L and S). Convergent primers were served as a negative control. dP divergent primers, cP convergent primers. **D** CircSPI1 isoforms were analyzed by RT-qPCR in THP-1 and NB4 cells. **E** The junction sites of circSPI1 isoforms were identified by sanger sequencing. All detected amplicons by divergent primers were subjected to TA clone, and then positive clones were performed sanger sequencing. **F** The relative expression levels of circSPI1 isoforms were analyzed in THP-1 and NB4 cells. The left two panels: RT-qPCR analysis measured the relative levels of five circSPI1s. The right two panels: TaqMan probe-based RT-qPCR assays determined the expression levels of circSPI1-Ex234-L and circSPI1-Ex234-S. All bar graphs represent the average of three independent replicates and error bars are SD, *P < 0.05, **P < 0.01, ***P < 0.001.

To systematically investigate the exon structure of circSPI1, we first used a pan-cancer circRNA compendium MiOncoCirc^14^ and identified five isoforms of circSPI1s originating from exons 2, 3, and 4 of the *SPI1* gene (Fig. 1A, lower left panel). Among them, one isoform is derived from exons 3 and 4 (circSPI1-Ex34); due to the alternative splicing of exon 2 on the linear *SPI1* gene (Fig. 1A, lower right panel), those originating from exons 2, 3, and 4 and from exons 2 and 3 produce two isoforms, respectively, named as circSPI1-Ex234-L and circSPI1-Ex234-S, and circSPI1-Ex23-L and circSPI1-Ex23-S. We next explored the expression pattern of circSPI1s in a variety of tissue-specific pan-cancer transcriptomes. We found that circSPI1s were mainly expressed in hematopoietic malignancies, e.g., acute myeloid leukemia (AML), chronic myeloid leukemia (CML), acute lymphocytic leukemia (ALL), chronic lymphocytic leukemia (CLL), myeloproliferative neoplasm (MPN), and multiple myeloma (MM), suggesting the hematopoietic lineage-specific pattern (Fig. 1B), similar to the lineage specificity observed for the parental *SPI1* gene. Next, we performed PCRs using a series of divergent and convergent primers in three AML cell lines, THP-1, NB4, and HL60. The results validated the existence of five circSPI1 isoforms in AML cells (Fig. 1C–E and Supplementary Fig. 1). For example, amplifying using divergent primers on exons 2 and 3 (Fig. 1C, left panel) resulted in two different bands, 300 bp and 140 bp, respectively, which remained stable after RNase R treatment (Fig. 1C, right panel; Fig. 1D; and Supplementary Fig. 1A, B). Sanger sequencing confirmed that the 300 bp band contained circSPI1-Ex234-L and circSPI1-Ex234-S, and the 140 bp band contained circSPI1-Ex23-L and circSPI1-Ex23-S, respectively (Fig. 1E, left four panels and Supplementary Fig. 1C). Similar results were obtained using divergent primers on exons 2 and 4, and on exons 3 and 4 (Fig. 1C–E and Supplementary Fig. 1). Next, we analyzed the relative expression level of the five isoforms by RT-qPCR. As shown in Fig. 1F (left two panels), the expression level of circSPI1-Ex234 (including both short and long isoforms) was remarkedly higher than that of circSPI1-Ex23 and circSPI1-Ex34. We further designed TaqMan probes specifically recognizing circSPI1-Ex234-L and circSPI1-Ex234-S and found that the expression of circSPI1-Ex234-S was substantially higher than that of circSPI1-Ex234-L (Fig. 1F, right two panels). The above finding motivated us to examine circSPI1-Ex234-S, which we called circSPI1 for simplicity to describe results in the following studies.

### CircSPI1 is specifically upregulated in AML

**Fig. 2:**
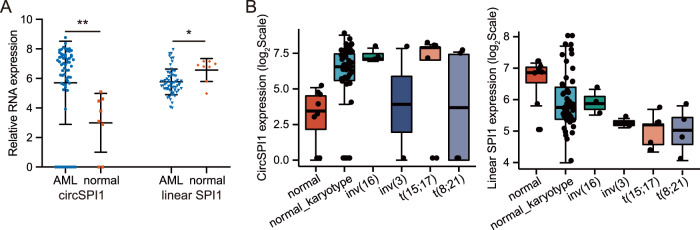
CircSPI1 is specifically upregulated in AML. **A** The relative expression levels of circSPI1 and linear *SPI1* in AML patients (*N* = 62) and healthy normal subjects (*N* = 8). **B** The expression levels of circSPI1 (left panel) and linear *SPI1* (right panel) in AML patients with different karyotypes. Ribo-zero RNA-sequencing data of 48 AML samples with normal karyotype, including 5 APL patients, 4 *t*(8; 21) translocation, 3 inv(16), 2 inv(3), and 8 normal individuals, were retrieved and analyzed. *P < 0.05, **P < 0.01.

We then investigated the expression level of circSPI1 in AML patients. We initially re-analyzed the ribo-zero RNA-sequencing data of 62 AML samples and 8 normal individuals using the uniform pipeline for fast and accurate detection of circRNAs^2,18,25,26^. Intriguingly, we observed the difference in the expression pattern between circSPI1 and linear *SPI1* in AML samples. As shown in Fig. 2A, the expression level of linear *SPI1* in AML was, as expected, lower than that in normal blood cells. In contrast, the expression level of circSPI1 in AML was surprisingly much higher than that in normal counterparts. AML is a group of heterogeneous disorders associated with distinct genetic aberrations, and the impairment of PU.1 is different in various subtypes, e.g., low expression in acute promyelocytic leukemia (APL) harboring *t*(15; 17)^5^, and low activity in AML with *t*(8; 21)^6^. We thus analyzed the expression levels of circSPI1 and linear *SPI1* in AML with diverse karyotypes and confirmed higher circSPI1 expression in AML, particularly in APL and AML with normal karyotype (Fig. 2B). The data suggested that circSPI1 was upregulated and showed a different expression pattern from parental gene *SPI1* in AML.

### CircSPI1 plays an oncogenic role in AML cells

**Fig. 3:**
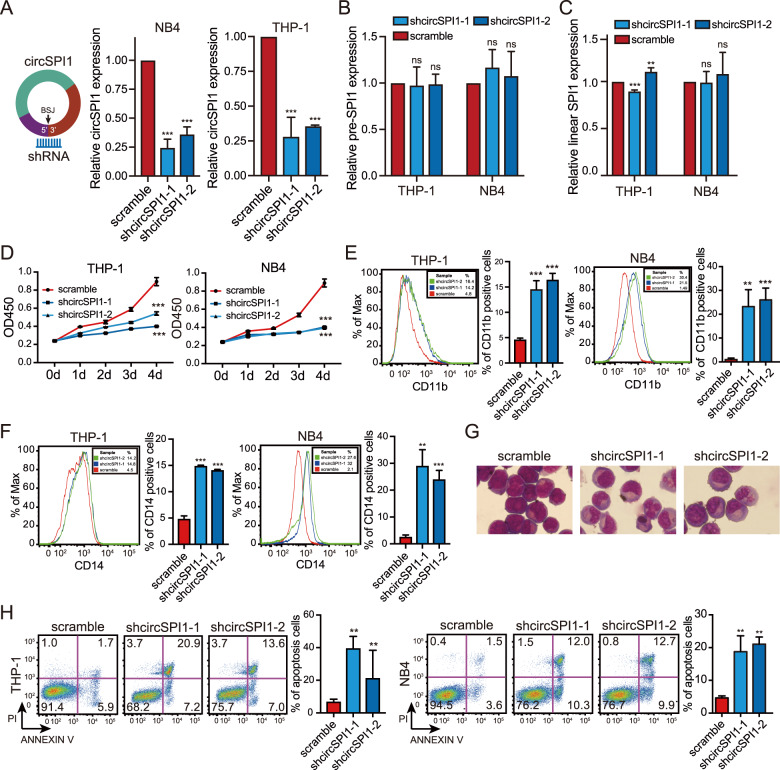
CircSPI1 plays an oncogenic role in AML cells. **A**–**C** The knockdown efficiency of shcircSPI1 was analyzed in THP-1 and NB4 cells. The expression of circSPI1 (**A**), pre-SPI1 (**B**), and linear *SPI1* (**C**) was demonstrated by RT-qPCR after infected with shcircSPI1 or scramble lentiviruses in THP-1 and NB4 cells. **D**–**H** CircSPI1 knockdown decreased proliferation, induced partial myeloid differentiation, and apoptosis in AML. THP-1 and NB4 cells were infected with shcircSPI1 or scramble lentiviruses. The cell proliferation ability was measured by the CCK8 assay 2 days after infection (**D**). Granulocytic (**E**) and monocytic (**F**) differentiation, cytomorphology (**G**), and apoptosis (**H**) were analyzed on day 4 after infection. Representative plots (left) and statistical percentage (right) of CD11b^+^, CD14^+^, or Annexin V^+^/PI^+/−^ cells are shown. Cell morphology was observed by Wright–Giemsa staining. All bar graphs represent the average of three independent replicates and error bars are SD, **P < 0.01, ***P < 0.001, ns no significance.

Given that high expression of circSPI1 was observed in AML patients, we then performed the knockdown experiments to explore the potential function of circSPI1 in AML. We designed two short hairpin RNAs (shRNAs) that specifically recognized the back-spliced junction site of circSPI1 and a nonspecifically scrambled shRNA as the negative control. Both shRNAs presented stronger knockdown effects to interfere with circSPI1 expression but did not affect the mRNA level of SPI1 (Fig. 3A–C). We then evaluated the influences of circSPI1 knockdown in both NB4 and THP-1 cells. Interestingly, we found that circSPI1 exerted an oncogenic function, distinct from its liner *SPI1* gene encoding PU.1 that is known as a tumor suppressor. Briefly, we demonstrated that silencing circSPI1 specifically decreased cell proliferation, induced partial myeloid differentiation and apoptosis of leukemic cells (Fig. 3D–H). First, CCK8 assays were performed to investigate cellular proliferation. As shown in Fig. 3D, the knockdown of circSPI1 significantly reduced the growth of THP-1 and NB4 cells. Second, the effect of circSPI1 on differentiation was determined using flow cytometry analysis. We found that circSPI1 knockdown significantly induced partial myeloid differentiation, evidenced by the increased expression of the cell surface marker CD11b for granulocytic differentiation (Fig. 3E) and the cell surface marker CD14 for monocytic differentiation (Fig. 3F). Consistently, cytomorphologic analysis by Wright–Giemsa staining further supported the differentiation phenotypes (Fig. 3G). Third, we detected the apoptosis of cells using Annexin V and PI and found that circSPI1 knockdown markedly increased the percentage of apoptosis in both THP-1 and NB4 cells (Fig. 3H).

### CircSPI1 antagonizes SPI1 to modulate myeloid differentiation

**Fig. 4:**
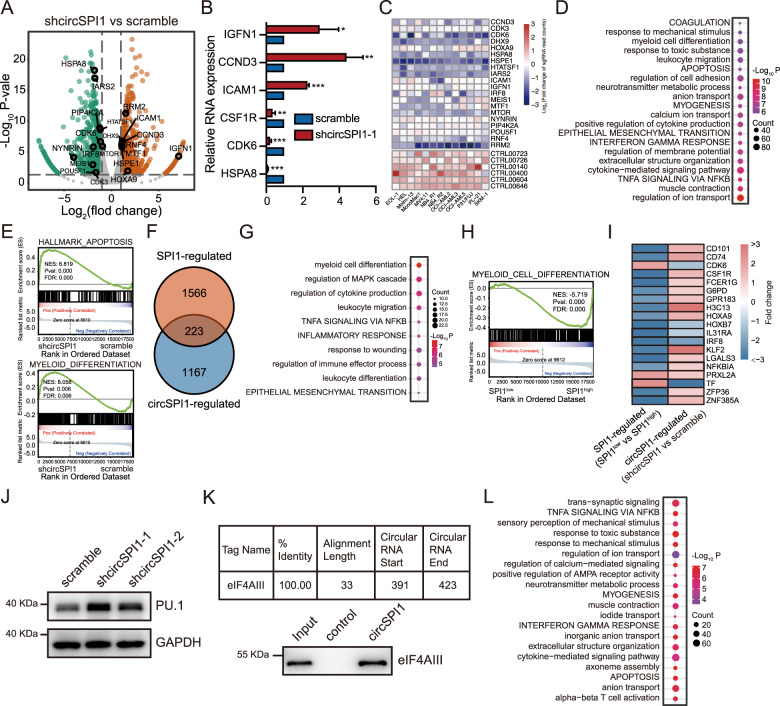
CircSPI1 antagonizes SPI1 to modulate myeloid differentiation. **A** Volcano plot of differentially expressed genes after circSPI1 knockdown. The vertical lines correspond to 2.0-fold up and down, and the horizontal line represents a *P* value of 0.05. **B** The expression changes of representative DEGs after circSPI1 knockdown were validated by RT-qPCR. **C** Representative DEGs upon circSPI1 knockdown were essential for the survival of AML cells. Normalized read counts of sgRNA in AML cells transfected with the CRISPR-cas9/sgRNA library before and after population doublings were showed. **D** Functional enrichment analysis showing significant GO terms and hallmark gene sets among circSPI1-regulated genes. **E** GSEA showing that differentiation and apoptosis were enriched using shcircSPI1-regulated genes. **F** The overlap of regulated genes between shcircSPI1 and shSPI1. **G** Top ten enriched functional terms for shared genes between circSPI1 and SPI1. **H** Differentiation was enriched using SPI1-regulated genes. **I** Heatmap showing that myeloid differentiation-associated genes were oppositely regulated by circSPI1 and SPI1. **J** The protein level of SPI1 was detected with or without circSPI1 knockdown. GAPDH served as the loading control. The original gels and three replicates are presented in Supplementary Fig. 3A. **K** CircSPI1 interacted with eIF4AIII in AML cells. The binding potential of circSPI1 with eIF4AIII was predicted by the Interactome database (upper panel). The interaction between circSPI1 and eIF4AIII was detected by western blotting combined with RNA pulldown (lower panel). Total lysates prepared from NB4 cells were subjected to pulldown with circSPI1 and negative control probes, followed by western blotting. The original gels and three replicates are presented in Supplementary Fig. 3B. **L** Functional enrichment of genes regulated by circSPI1 but not SPI1 was summarized using annotated GO terms and hallmark gene sets. All bar graphs represent the average of three independent replicates and error bars are SD, *P < 0.05, **P < 0.01, ***P < 0.001.

To determine how circSPI1 affects functional pathways, we performed RNA-seq of NB4 cells with and without circSPI1 knockdown. We found 1390 differentially expressed genes (DEGs) upon circSPI1 knockdown, including 907 upregulated and 483 downregulated (Fig. 4A and Supplementary Table 2). The data were confirmed by RT-qPCR (Fig. 4B). Noteworthy, a fair number of these genes have been reported to contribute to leukemogenesis or maintenance of AML cells, e.g., IRF8^27^, ICAM1^28^, HOXA9^29^, and CDK6^30^, which was validated by the genome-wide CRISPR-Cas9 screening data of AML cell lines^31^ (Fig. 4C). Functional enrichment analysis revealed that circSPI1-regulated genes were mainly involved in apoptosis, myeloid differentiation as well as immune-related pathways (Fig. 4D). GSEA confirmed the association of circSPI1-regulated genes with the functional categories of differentiation and apoptosis (Fig. 4E). The transcriptome data supported the phenotypic observations upon circSPI1 knockdown in AML cells (Fig. 3D–H).

Given the antagonistic effects in myeloid differentiation observed in the functional experiment of PU.1 and circSPI1, we next asked whether circSPI1 was involved in the modulation of PU.1-mediated myeloid differentiation. To test this speculation, we first generated SPI1-regulated genes in AML patients. We retrieved the gene expression profile of AML from the TCGA dataset, divided the samples into SPI1^high^ and SPI1^low^ groups according to the *SPI1* expression levels (Supplementary Fig. 2, 10% quantile vs. 90% quantile). We obtained 1789 DEGs potentially influenced by SPI1 by comparing the SPI1^low^ group to the SPI1^high^ group, including 855 SPI1-upregulated and 934 SPI1-downregulated (Supplementary Table 3). The well-known SPI1 target genes, such as M-CSF receptor *CSF1R*, *IRF8*, *HCK*, were indeed among them. We then compared circSPI1-regulated genes and SPI1-regulated genes and identified 223 genes commonly regulated by circSPI1 and SPI1 (Fig. 4F). Functional enrichment analysis of common genes revealed that myeloid cell differentiation was the most top enriched pathway (Fig. 4G), highlighting their common involvement in modulating myeloid cell differentiation. Interestingly, however, GSEA illustrated that this list of SPI1-regulated (SPI1^low^ vs. SPI1^high^) genes tended to be negatively enriched in the category of myeloid cell differentiation (Fig. 4H), consistent with the reported function of SPI1 in AML. The genes enriched in the myeloid cell differentiation showed an opposite expression pattern (Fig. 4I). These results suggested that circSPI1 may be involved in the negative control for SPI1 expression in modulating myeloid cell differentiation.

Since circSPI1 had no impact on the mRNA expression of SPI1 (Fig. 3C), we then examined whether circSPI1 exerted an impact on the protein level of SPI1 (PU.1). We performed western blotting and found that the knockdown of circSPI1 indeed resulted in the increase of the PU.1 protein level (Fig. 4J and Supplementary Fig. 3A). The result suggested that circSPI1 might repress PU.1 translation. To explore the mechanisms of the translational interference by circSPI1, we utilized the web tool CircInteractome^32^ to search for the translation associated proteins that interacted with circSPI1 and found that the translation initiation factor eIF4AIII was a promising candidate (Fig. 4K, upper panel). To validate the prediction result, we then performed RNA pulldown assay using circSPI1 probe and found eIF4AIII strongly bound to circSPI1, as compared with negative control (Fig. 4K, lower panel and Supplementary Fig. 3B).

The comparison in Fig. 4F also showed that the majority of the circSPI1-regulated genes were not overlapped with SPI1-regulated genes, suggesting that circSPI1 also exerted distinct functions uncoupled with SPI1 in AML. We, therefore, performed functional enrichment analysis of 1176 genes that were specifically regulated by circSPI1, revealing the enrichment of ion transport, myogenesis, immune response, as well as cell apoptosis (Fig. 4L). Combining our above functional experiments, we concluded that circSPI1 could lead to AML phenotypes by antagonizing PU.1 and other potential mechanisms that need to clarify further.

### CircSPI1 interacts with microRNAs to regulate functional signaling pathways

**Fig. 5:**
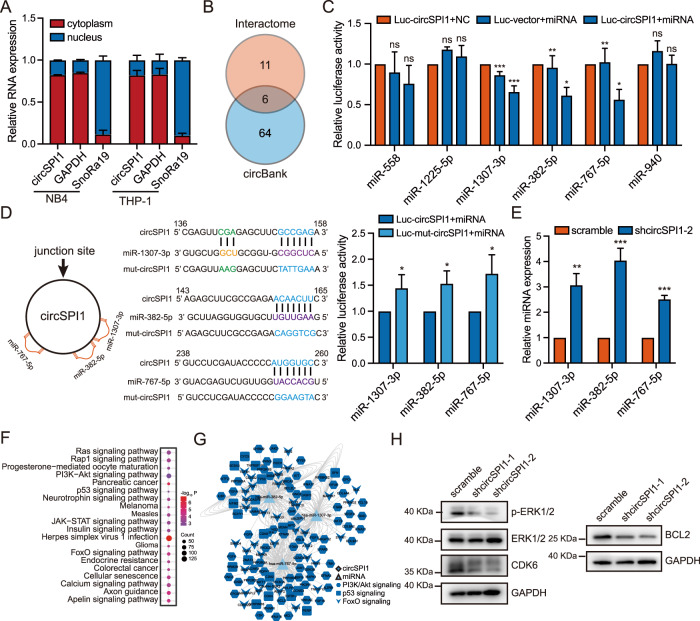
CircSPI1 interacts with microRNAs to regulate apoptosis functional signaling pathways. **A** The subcellular localization of circSPI1 was analyzed by RT-qPCR. **B** Potential targeted miRNAs of circSPI1 were predicted by the circBank and Interactome databases. **C** Luciferase activity assay detected the miRNAs bound by circSPI1. HEK-293T cells were transfected with the circSPI1 luciferase reporter plasmid and predicted miRNA mimics. The luciferase activity was detected 24 h after transfection. **D** Luciferase activity of mutant circSPI1 was analyzed in HEK-293T cells transfected with the three miRNA mimics. The schematic diagram delineated putative binding sites of miR-1307-3p, miR-382-5p, and miR-767-5p on circSPI1 (left two panels). The luciferase activity was detected 24 h after transfection (right panel). **E** The expression levels of the three miRNAs were detected with or without circSPI1 knockdown by RT-qPCR**. F** Enriched KEGG terms for the three miRNA-regulated genes. **G** Global view of the circSPI1 and three miRNAs-associated ceRNA network. **H** The protein levels of apoptosis-related factors were detected with or without circSPI1 knockdown by western blotting. The original gels and three replicates are presented in Supplementary Fig. 4. All bar graphs represent the average of three independent replicates and error bars are SD, *P < 0.05, **P < 0.01, ***P < 0.001, ns no significance.

We next explored the other potential mechanisms underlying circSPI1 action, in particular through identifying microRNAs (miRNAs), since circRNAs may function by interacting with miRNAs^15,33^. To address whether circSPI1 could interact with miRNA in AML, we first analyzed its subcellular localization and found that circSPI1 was mainly located in the cytoplasm, suggesting that cytoplasm may be the main arena and can interact with miRNA (Fig. 5A). We next predicted the potential microRNAs bound by circSPI1 using the Interactome^32^ and circBank databases^34^ and identified six highly confident miRNAs (Fig. 5B). To determine which miRNA(s) were bound by circSPI1, we then performed the luciferase experiments using the mimics of these six miRNAs. As shown in Fig. 5C, miR-1307-3p, miR-382-5p, and miR-767-5p were significantly bound by circSPI1. Figure 5D (right two panels) illustrated the miRNA-binding sites on circSPI1. Furthermore, when the binding sites on circSPI1 were mutated, the decreased luciferase activity in wild-type circSPI1 was reverted (Fig. 5D, right panel). We also found that the expression of the three miRNAs was increased after circSPI1 knockdown (Fig. 5E).

We next predicted the targets of these three miRNAs using a comprehensive archive, miRWalk^35^, which provides the biggest and newest available collection of predicted and experimentally verified miRNA-target interactions. Using highly confident binding sites on these three miRNAs (Supplementary Table 4), we performed KEGG pathway enrichment analysis and found that these three miRNAs-mediated target genes were significantly involved in cancer pathways, including Ras, PI3K-Akt, p53, JAK-STAT, and FoxO signaling (Fig. 5F). Accumulating evidence has demonstrated that p53^36^, PI3K-Akt^37^, and FoxO^38^ play important roles in diverse intracellular signaling pathways, including cell growth inhibitory and/or apoptosis. We further analyzed the enriched genes in these pathways and constructed a competing endogenous RNA (ceRNA) network. As shown in Fig. 5G, a fair of apoptosis-associated genes, e.g., BCL2^39^, were also regulated by circSPI1 through interacting with miRNAs. We further conducted western blotting and found that BCL2, CDK6, and p-ERK1/2, which were involved in apoptosis, were decreased upon circSPI1 knockdown (Fig. 5H and Supplementary Fig. 4). Taken together, these results suggested that circSPI1 could function by interacting with miR-1307-3p, miR-382-5p, and miR-767-5p to participate in important signaling pathways, including apoptosis.

### CircSPI1-mediated gene network is associated with the prognosis of AML patients

**Fig. 6:**
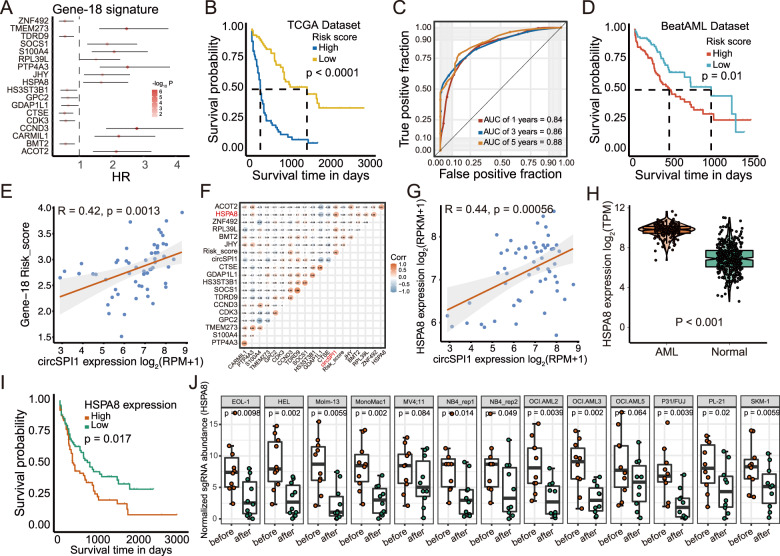
CircSPI1-mediated gene network is associated with prognosis of AML patients. **A** The hazard ratios of the LASSO selected 18 genes. **B** Kaplan–Meier curve for the overall survival (OS) of AML patients classified by the median of Gene-18 risk score. **C** Receiver-operating characteristic (ROC) curve of the Gene-18 risk score. **D** Kaplan–Meier curve for the overall survival (OS) of AML patients classified by the Gene-18 risk score in the validated set. **E** The correlation of circSPI1-regulated 18-gene risk score and circSPI1 expression. **F** Correlation matrix of circSPI1 and circSPI1-regulated 18 genes. RNA quantification analysis was performed on the transcriptomic data of AML patients with enough sequencing depth that enabled the identification of both linear *SPI1* and circSPI1. The Pearson method was used to assess the correlations among circSPI1 and circSPI1-regulated genes. **G** The correlation of *HSPA8* and circSPI1 expression. **H** Expression levels of *HSPA8* were analyzed in AML patients and normal subjects. **I** Kaplan–Meier curve for the overall survival (OS) of AML patients with high or low *HSPA8* expression levels. **J** HSPA8 was essential for the survival of AML cells. Normalized read counts of sgRNA in AML cells transfected with the CRISPR-cas9/sgRNA library before and after population doublings are shown.

Given the oncogenic role of circSPI1 in AML cells, we proceeded to explore whether circSPI1-regulated genes have the potential to be associated with the clinical outcome of AML patients. Indeed, many poor prognosis-related genes, e.g., *CDK3*, *IRF8*, *ICAM1*, were differentially regulated by circSPI1. We thus used a univariate Cox regression coupled with a machine-learning method and identified eighteen genes among circSPI1-regulated genes (Fig. 6A). As shown in Fig. 6B, C, these circSPI1-regulated 18 genes were capable of distinguishing the high-risk AML group (shorter OS) from the low-risk group (longer OS) (*P* < 0.0001). The significant prognostic value was also confirmed in the validation dataset of BeatAML (Fig. 6D). Further univariate and multivariable Cox regression analysis indicated that the score formed by circSPI1-regulated 18 genes was independent of the patient age, cytogenetics risk, and ELN2017 risk stratification, as well as the status of FLT3 and NPM1 mutations (Supplementary Table 5). Next, to investigate the prognostic value of circSPI1 in AML, we performed RNA quantification analysis on the transcriptomic data of AML patients with sufficient sequencing depth that enabled the identification of both linear *SPI1* and circSPI1. We calculated the risk score of these circSPI1-regulated 18 genes for each patient and correlated it with circSPI1 expression. As shown in Fig. 6E, circSPI1 expression level was positively correlated with the risk score of these 18 genes, with a higher expression, suggesting a more severe risk prognosis. Among them, *HSPA8* was the top one correlated with circSPI1 (Fig. 6F), which therefore was selected for further validation. *HSPA8* expression was positively correlated with circSPI1 expression (Fig. 6G). Differential expression study showed that *HSPA8* was upregulated in AML patients compared with normal subjects (Fig. 6H), and higher expression suggested an adverse prognosis for AML (Fig. 6I). Using CRISPR-based screening data of 12 AML cell lines, we found that *HSPA8* was essential for the survival of AML cells (Fig. 6J), supporting the functional importance of *HSPA8* for the maintenance of AML. Collectively, these results illustrated that circSPI1 could serve as a prognostically important biomarker for AML.

## Discussion

In this study, we identified and characterized circSPI1 generated from the locus of SPI1 that encodes the myeloid transcriptional master regulator PU.1. We demonstrated that circSPI1 was frequently upregulated and functioned as an oncogene, distinct from linear *SPI1*-encoding PU.1. We revealed that circSPI1 regulated myeloid differentiation by antagonizing SPI1 and controlled proliferation and apoptosis through interacting with miRNAs. Our findings reinforce the importance of circRNAs, especially those derived from the genetic loci encoding hematopoietic transcription factors.

Our finding uncovers circSPI1 as a novel layer of the information on the *SPI1* locus in leukemogenesis. We demonstrated that circSPI1 acted as an oncogene to control cellular proliferation, myeloid differentiation, and apoptosis in AML, which was distinct from linear *SPI1* encoded PU.1, a well-known tumor suppressor^40^. The impairment of PU.1 expression or activity has been commonly observed in AML. The opposite function of circSPI1 and SPI1 was consistent with a recent finding showing that circPOK acts as a proto-oncogenic noncoding RNA antithetically and independently to its linear partner, the tumor suppressor gene Pokmon^41^. We call on more efforts to characterize the diverse roles of circRNAs in AML pathology.

We demonstrated that circSPI1 negatively regulated the protein level of SPI1 to orchestrate myeloid differentiation. The current knowledge of the action of circRNAs mainly focuses on the parental-independent manner^42,43^ or the positive control of its linear RNA^44^. Our finding suggested a new role of circRNAs through the ability to negatively control the expression of parental coding genes. The precise expression levels of PU.1 are critical for hematopoietic development, and, if perturbed, even moderate decreases can lead to AML^9,10^. PU.1 has been reported to be strictly controlled to a proper level by multiple mechanisms, including the positive auto-regulatory loop-mediated through an upstream regulatory element^11,12^, the balance of its functional sense, and antisense RNAs from the *SPI1* locus^13^ and microRNAs^7,45^. Our finding suggests that circRNAs may serve as an important factor ensuring the tight regulation of PU.1 in hematopoiesis and AML development.

It deserved mentioning that circSPI1 was frequently upregulated in AML and circSPI1-regulated genes were associated with the prognosis of AML patients. This implicated that circSPI1 and its regulated genes might be potential biomarkers for AML diagnosis and therapy due to remarkable features of circRNAs, e.g., high abundance in saliva^46^, exosomes^47^, and clinical standard blood samples^26,48^, and stability caused by the covalently closed loop structures^49^. More investigations, however, are needed to test their power as a biomarker for AML.

Last but not least, we found that the upregulated expression pattern of circSPI1 was inverse with linear *SPI1* in AML. This is probably due to the biogenesis or post-transcriptional regulatory mechanisms not shared by linear and circular genes, such as introns of linear transcripts. Indeed, the PML/RARα fusion protein can bind to the third intron of *SPI1* and, in turn decrease the transcription and translation of *SPI1*^13,50^. In contrast, RNA-binding proteins involved in circRNAs biogenesis are frequently dysregulated in AML. In future studies, the regulatory mechanisms between circSPI1 and linear *SPI1* require to be fully addressed.

## Supplementary information

Supplementary Figures

Supplementary Table 1

Supplementary Table 2

Supplementary Table 3

Supplementary Table 4

Supplementary Table 5

## Data Availability

All codes are available upon reasonable request.
