# Supplementary material for: CircSPI1 acts as an oncogene in acute myeloid leukemia through antagonizing SPI1 and interacting with microRNAs
Source: Cell Death Dis. 2021 Mar 19;12(4):297. doi: 10.1038/s41419-021-03566-2 (PMC7979773; doi:10.1038/s41419-021-03566-2)
Supplement: Supplementary file 1 — Supplementary Figures [file 41419_2021_3566_MOESM1_ESM.docx]

**Supplementary Figures**


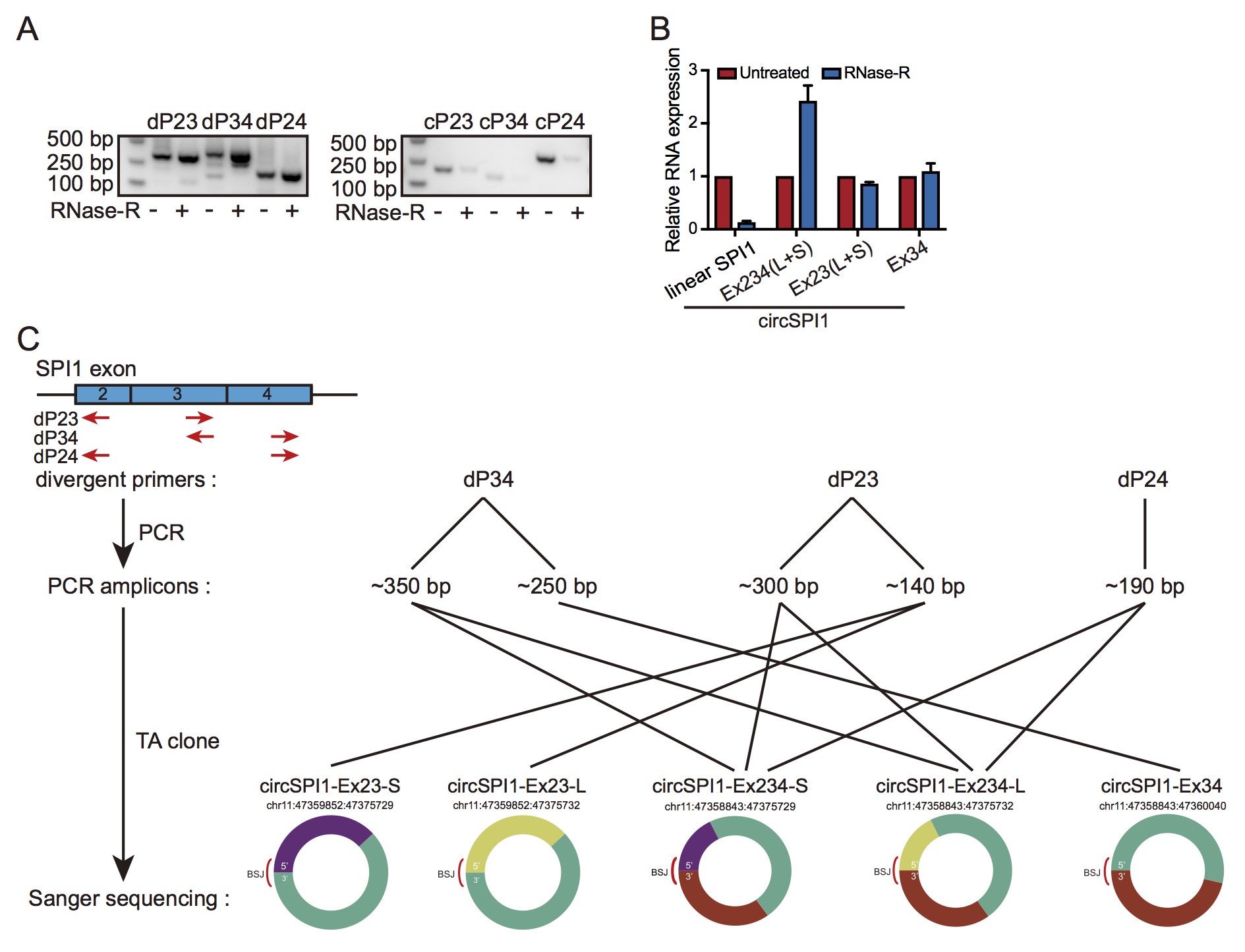


**Supplementary Fig. 1** **Identification and characterization of circSPI1 in acute myeloid leukemia**

**A**. The expression of circSPI1s was validated with divergent and convergent primers in HL60 cells. Two major bands produced by the divergent primer pair dP23 corresponded to circSPI1-Ex234 (L and S) and circSPI1-Ex23 (L and S), respectively. Two major bands also produced by the divergent primer pair dP34 corresponded to circSPI1-Ex234 (L and S) and circSPI1-Ex34. One band produced by dP24 corresponded to circSPI1-Ex234 (L and S). Convergent primers were served as negative control. dP: divergent primers cP: convergent primers. **B**. CircSPI1 isoforms were analyzed by RT-qPCR in HL60 cells. **C**. Schematic overview showed the process of TA clone to identity the junction sites of detected PCR amplicons.


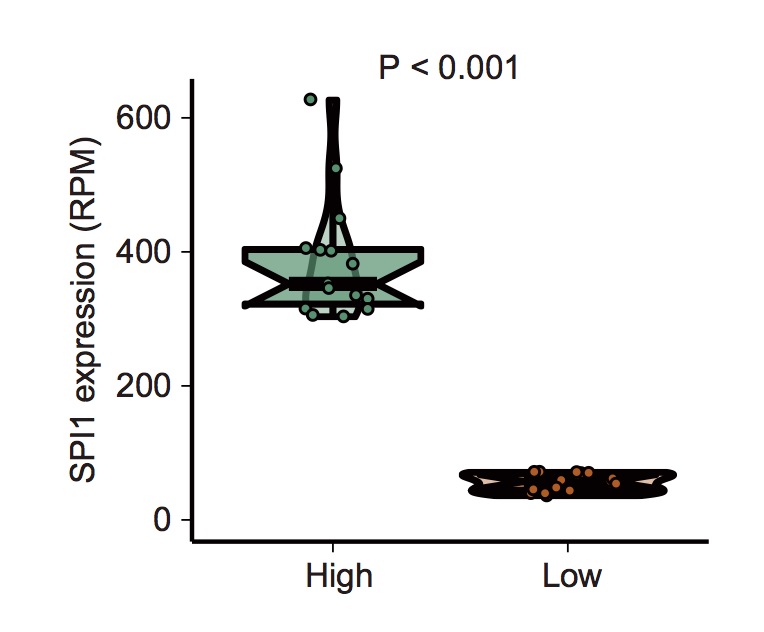


**Supplementary Fig. 2 Boxplot of SPI1 expression in TCGA AML patients.**

The AML samples were divided into SPI1^high^ (N=15) and SPI1^low^ (N=15) groups according to the *SPI1* expression levels (10% quantile vs 90% quantile).


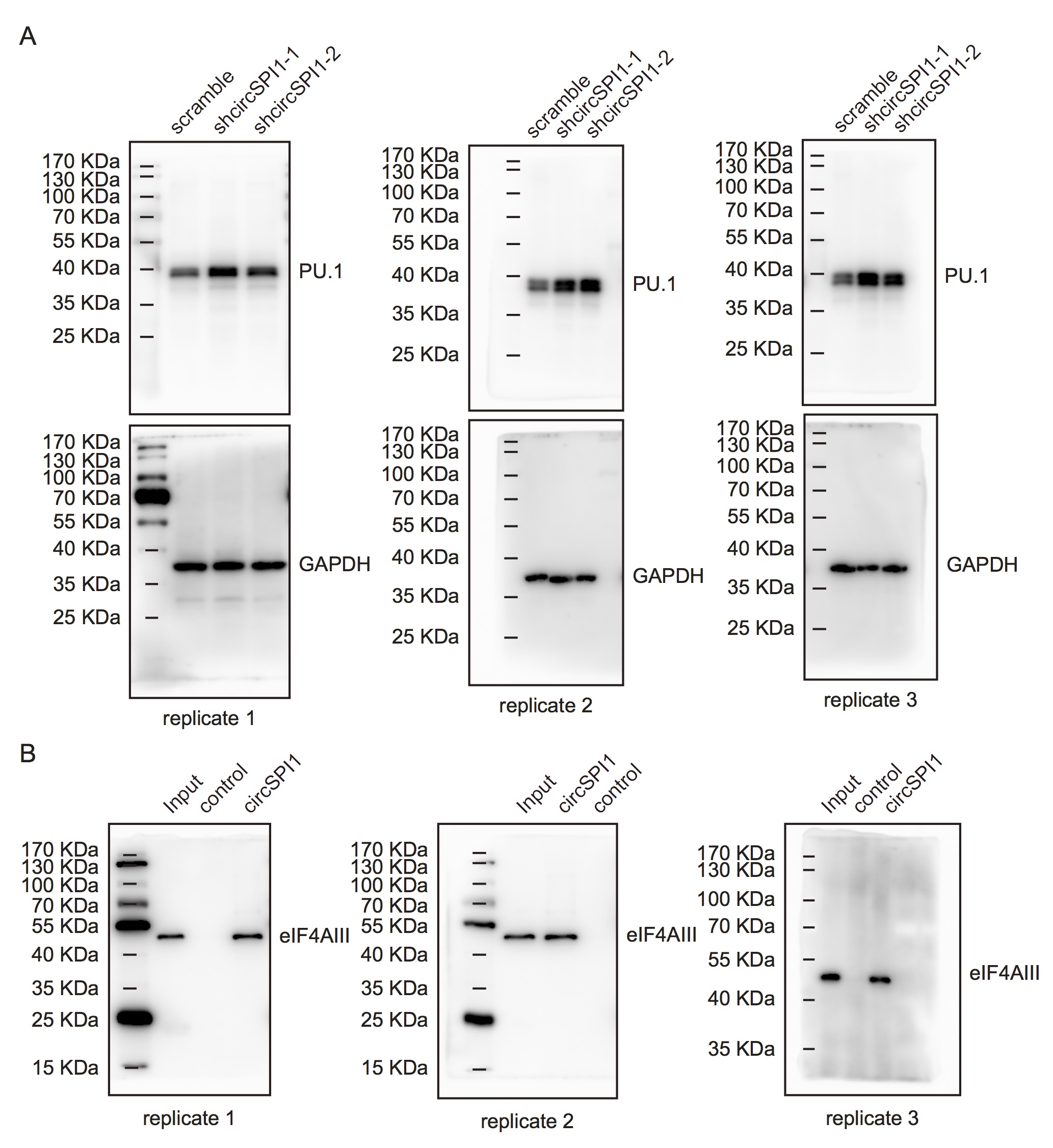


**Supplementary Fig. 3 Original gels of western blotting in this study.**

**A**. The protein level of SPI1 was detected with or without circSPI1 knockdown for three replicates with original gels. GAPDH served as the loading control. **B**. The interaction between circSPI1 and eIF4AIII was detected by western blotting combined with RNA pulldown for three replicates with original gels.


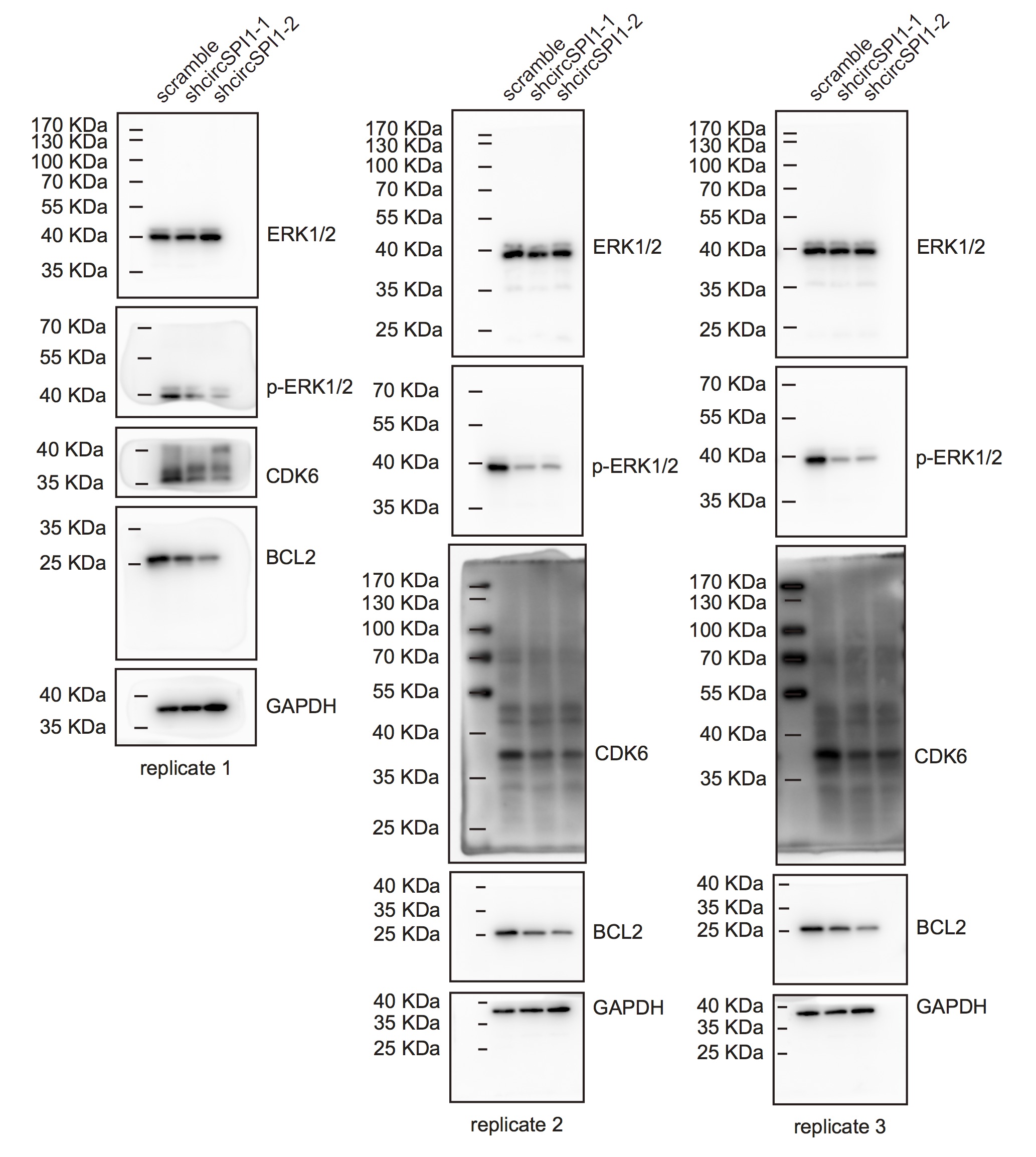


**Supplementary Fig. 4** The protein levels of apoptosis related factors were detected with or without circSPI1 knockdown by western blotting for three replicates with original gels.
